# Supplementary material for: SARS‐CoV‐2 structural coverage map reveals viral protein assembly, mimicry, and hijacking mechanisms
Source: Mol Syst Biol. 2021 Sep 14;17(9):e10079. doi: 10.15252/msb.202010079 (PMC8438690; doi:10.15252/msb.202010079)
Supplement: Supplementary file 1 — Appendix [file MSB-17-e10079-s006.pdf]

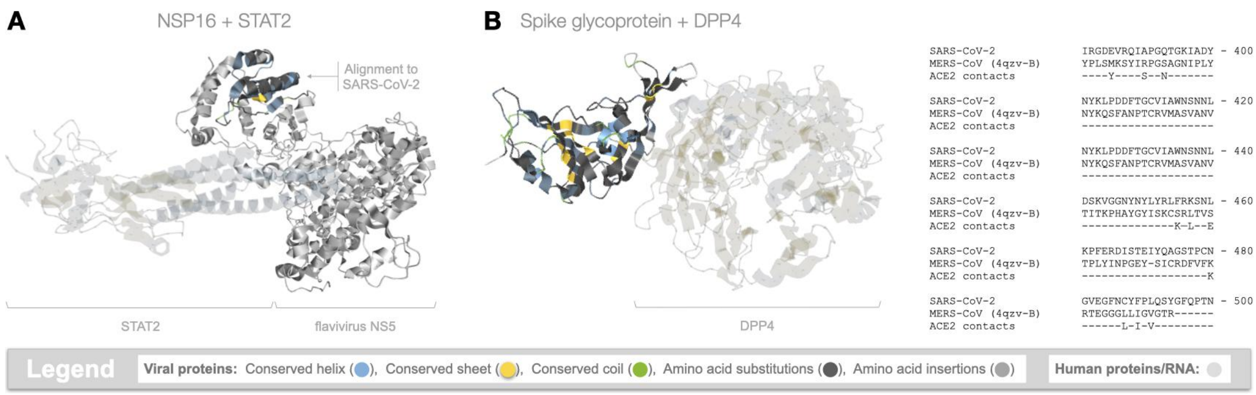

Appendix Figure S1. Assessing structural evidence for STAT2 and DPP4 hijacking

These matching structures show putative interactions between viral and human proteins, but detailed analysis finds insufficient evidence that these proteins are hijacked by SARS-CoV-2. The structures are shown with Aquaria's default coloring scheme which, for viral proteins, highlights secondary structure as well as any amino acid substitutions and insertions in the alignment the SARS-CoV-2 sequences; human proteins are rendered as semi-transparent.

A. Hijacking of STAT2 was shown in only one matching structure that was determined using a distantly related flavivirus protein called NS5 (Wang et al., 2020). Mapping the SARS-CoV-2 NSP16 sequence onto this structure (P0DTD1/6wcz) reveals that the alignment has moderate significance (E = 10^{-16}), low pairwise identity (13%), and does not include any of the residues that contact STAT2. Thus, we conclude that this matching structure does not provide evidence of hijacking of STAT2 by SARS-CoV-2.

B. Hijacking of DPP4 was shown in only two matching structures, both determined using the spike glycoprotein from MERS-CoV (Lu et al., 2013; Wang et al., 2014). Mapping the SARS-CoV-2 spike glycoprotein sequence onto these structures reveals that the alignment has high significance (E ~ 10^{-46}); however, for the structure shown here (P0DTC2/4qzv), the overall pairwise similarity is low (21%), with no sequence identities amongst the 10 residue positions that directly contact DPP4. Thus, we concluded that these matching structures do not provide strong evidence of hijacking of STAT2 by SARS-CoV-2.

Made using Aquaria and edited in Keynote.

References

Lu, G., Hu, Y., Wang, Q., Qi, J., Gao, F., Li, Y., Zhang, Y., Zhang, W., Yuan, Y., Bao, J., Zhang, B., Shi, Y., Yan, J., Gao, G.F., 2013. Molecular basis of binding between novel human coronavirus MERS-CoV and its receptor CD26. Nature 500, 227–231. https://doi.org/10.1038/nature12328

Wang, B., Thurmond, S., Zhou, K., Sánchez-Aparicio, M.T., Fang, J., Lu, J., Gao, L., Ren, W., Cui, Y., Veit, E.C., Hong, H., Evans, M.J., O'Leary, S.E., García-Sastre, A., Zhou, Z.H., Hai, R., Song, J., 2020. Structural basis for STAT2 suppression by flavivirus NS5. Nat. Struct. Mol. Biol. 27, 875–885. https://doi.org/10.1038/s41594-020-0472-y

Wang, Q., Qi, J., Yuan, Y., Xuan, Y., Han, P., Wan, Y., Ji, W., Li, Y., Wu, Y., Wang, J., Iwamoto, A., Woo, P.C.Y., Yuen, K.-Y., Yan, J., Lu, G., Gao, G.F., 2014. Bat Origins of MERS-CoV Supported by Bat Coronavirus HKU4 Usage of Human Receptor CD26. Cell Host Microbe 16, 328–337. https://doi.org/10.1016/j.chom.2014.08.009

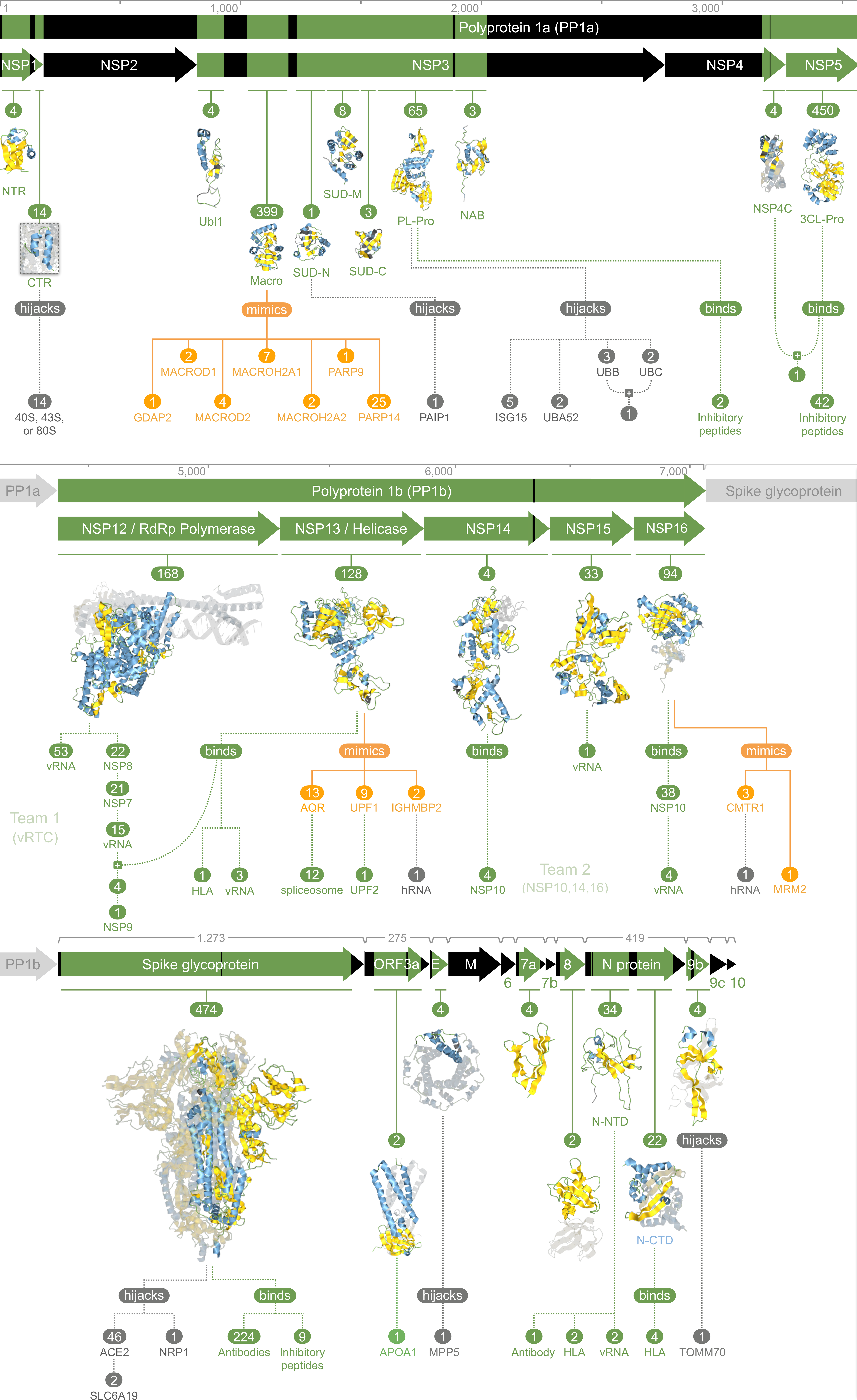

Legend: SARS-CoV-2 structural coverage map

- Known structure
- Viral protein sequence
- Unknown structure
- Number of structures
- Substitutions
- Helix, Coil, Sheet — Conserved residues
- Indicates this is part of a much larger structure
- Other proteins or RNA
- XYZ — Name of protein domain
- hijacks — Structures show viral proteins hijacking human proteins
- mimics — Structures show human proteins that the viral protein may mimic
- binds — Structures show binding to viral proteins, HLA, or inhibitory peptides
- 40 Viral protein A — 40 structures show binding to viral protein A
- 8 Viral protein B — Of the above 40 structures, eight show binding to viral protein B
- One structure shows binding between the two adjacent proteins

Abbreviations:

40S: small ribosomal subunit

43S: pre-initiation complex

80S: ribosome

E: viral envelope protein

HLA: human leukocyte antigen

hRNA: host RNA

M: matrix glycoprotein

N: nucleocapsid protein

vRNA: viral RNA

vRTC: viral replication and translation complex

Data sources:

- The coverage map was based on Swiss-Prot sequences available on March 11, 2021.
- These sequence have been aligned onto all related PDB structures available on March 27, 2021.
- A total of 2,060 related structures were found.
- 1,180 of these structures were found from database cross-references in PDB entries.
- An additional 880 related structural models were found using HHBlits with cut-offs of either $E \leq 10^{-10}$ or pairwise identity ≥ 90%.

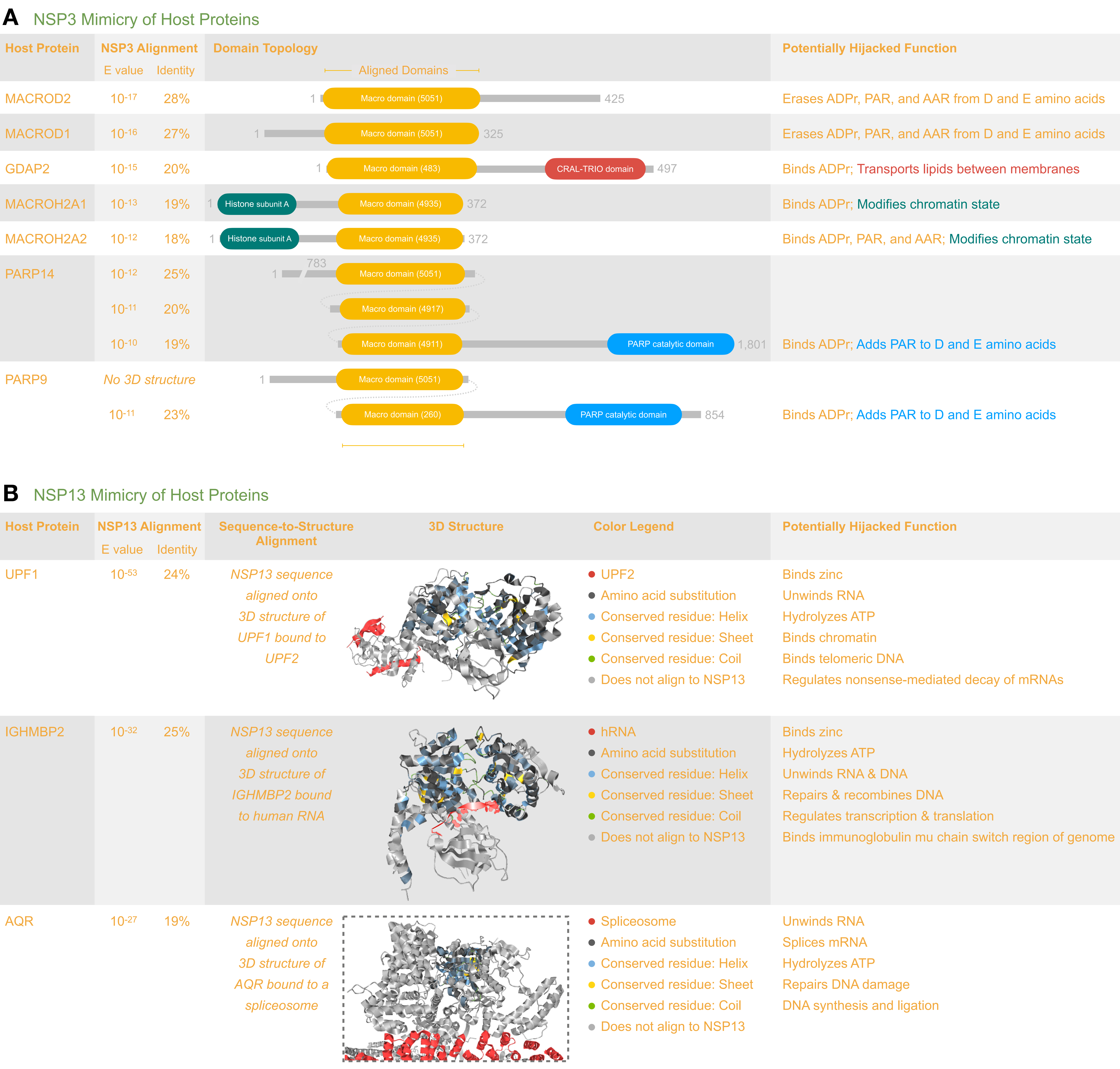

A NSP3 Mimicry of Host Proteins
Host Protein
NSP3 Alignment
E value
Identity
Domain Topology
Aligned Domains
Potentially Hijacked Function
MACROD2 | 10-17 | 28%
1 Macro domain (5051) 425
Erases ADPr, PAR, and AAR from D and E amino acids
MACROD1 | 10-16 | 27%
1 Macro domain (5051) 325
Erases ADPr, PAR, and AAR from D and E amino acids
GDAP2 | 10-15 | 20%
1 Macro domain (483) CRAL-TRIO domain 497
Binds ADPr; Transports lipids between membranes
MACROH2A1 | 10-13 | 19%
1 Histone subunit A Macro domain (4935) 372
Binds ADPr; Modifies chromatin state
MACROH2A2 | 10-12 | 18%
1 Histone subunit A Macro domain (4935) 372
Binds ADPr, PAR, and AAR; Modifies chromatin state
PARP14 | 10-12 | 25%
1 783 Macro domain (5051)
10-11 | 20%
Macro domain (4917)
10-10 | 19%
Macro domain (4911) PARP catalytic domain 1,801
Binds ADPr; Adds PAR to D and E amino acids
PARP9 | No 3D structure
1 Macro domain (5051)
10-11 | 23%
Macro domain (260) PARP catalytic domain 854
Binds ADPr; Adds PAR to D and E amino acids
B NSP13 Mimicry of Host Proteins
Host Protein
NSP13 Alignment
E value
Identity
Sequence-to-Structure Alignment
3D Structure
Color Legend
Potentially Hijacked Function
UPF1 | 10-53 | 24%
NSP13 sequence aligned onto 3D structure of UPF1 bound to UPF2
UPF2
Amino acid substitution
Conserved residue: Helix
Conserved residue: Sheet
Conserved residue: Coil
Does not align to NSP13
Binds zinc
Unwinds RNA
Hydrolyzes ATP
Binds chromatin
Binds telomeric DNA
Regulates nonsense-mediated decay of mRNAs
IGHMBP2 | 10-32 | 25%
NSP13 sequence aligned onto 3D structure of IGHMBP2 bound to human RNA
hRNA
Amino acid substitution
Conserved residue: Helix
Conserved residue: Sheet
Conserved residue: Coil
Does not align to NSP13
Binds zinc
Hydrolyzes ATP
Unwinds RNA & DNA
Repairs & recombines DNA
Regulates transcription & translation
Binds immunoglobulin mu chain switch region of genome
AQR | 10-27 | 19%
NSP13 sequence aligned onto 3D structure of AQR bound to a spliceosome
Spliceosome
Amino acid substitution
Conserved residue: Helix
Conserved residue: Sheet
Conserved residue: Coil
Does not align to NSP13
Unwinds RNA
Splices mRNA
Hydrolyzes ATP
Repairs DNA damage
DNA synthesis and ligation

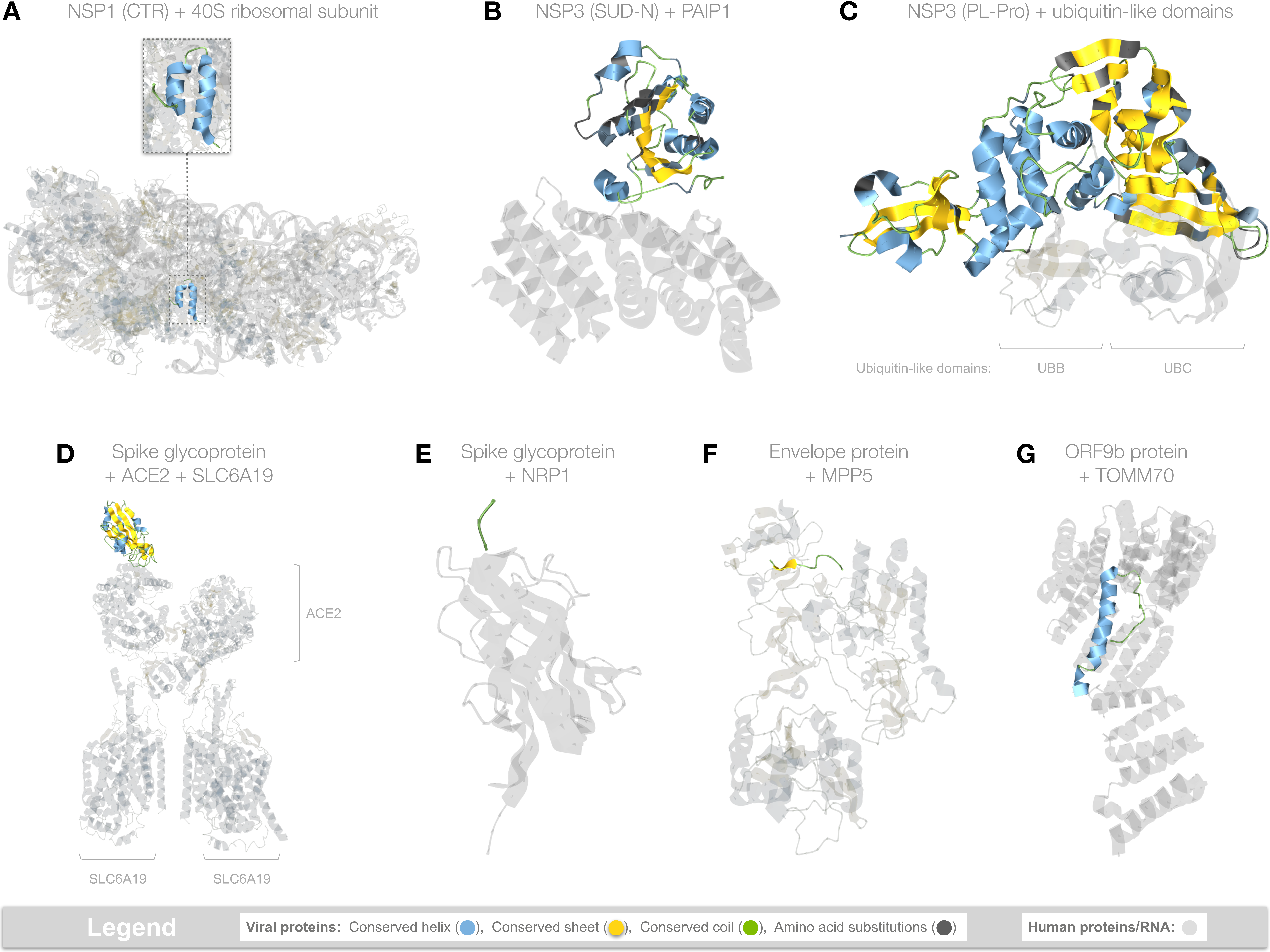
A
NSP1 (CTR) + 40S ribosomal subunit
B
NSP3 (SUD-N) + PAIP1
C
NSP3 (PL-Pro) + ubiquitin-like domains
Ubiquitin-like domains:
UBB
UBC
D
Spike glycoprotein
+ ACE2 + SLC6A19
ACE2
SLC6A19
SLC6A19
E
Spike glycoprotein
+ NRP1
F
Envelope protein
+ MPP5
G
ORF9b protein
+ TOMM70
Legend
Viral proteins: Conserved helix ( ), Conserved sheet ( ), Conserved coil ( ), Amino acid substitutions ( )
Human proteins/RNA:

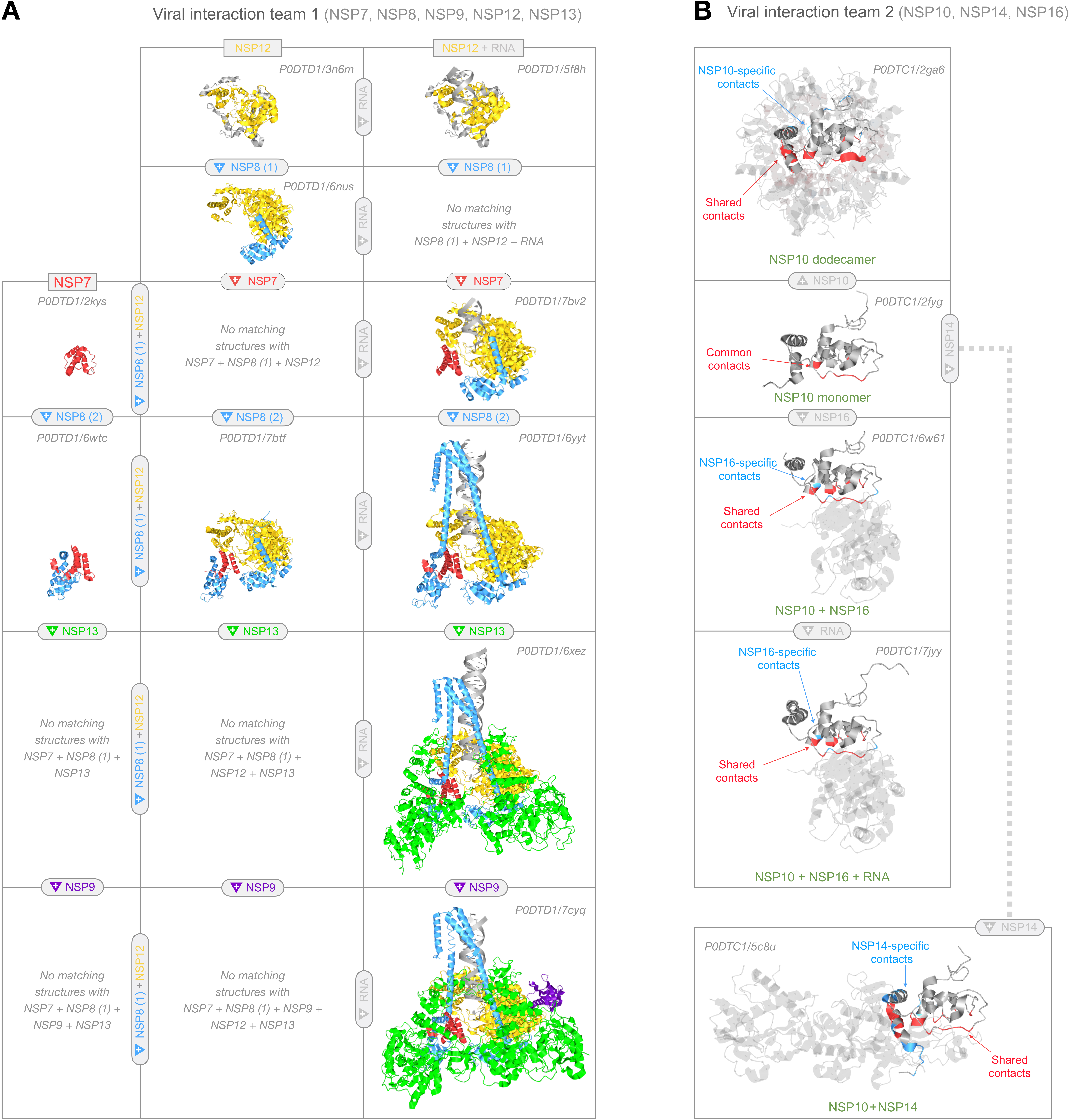

A
Viral interaction team 1 (NSP7, NSP8, NSP9, NSP12, NSP13)
NSP12
NSP12 + RNA
P0DTD1/3n6m
P0DTD1/5f8h
RNA
NSP8 (1)
P0DTD1/6nus
No matching structures with NSP8 (1) + NSP12 + RNA
NSP7
P0DTD1/2kys
NSP8 (1) + NSP12
No matching structures with NSP7 + NSP8 (1) + NSP12
P0DTD1/7bv2
NSP8 (2)
P0DTD1/6wtc
P0DTD1/7btf
P0DTD1/6yyt
NSP13
P0DTD1/6xez
No matching structures with NSP7 + NSP8 (1) + NSP13
No matching structures with NSP7 + NSP8 (1) + NSP12 + NSP13
NSP9
P0DTD1/7cyq
No matching structures with NSP7 + NSP8 (1) + NSP9 + NSP13
No matching structures with NSP7 + NSP8 (1) + NSP9 + NSP12 + NSP13
B
Viral interaction team 2 (NSP10, NSP14, NSP16)
NSP10-specific contacts
P0DTC1/2ga6
Shared contacts
NSP10 dodecamer
NSP10
P0DTC1/2fyg
Common contacts
NSP14
NSP10 monomer
NSP16
P0DTC1/6w61
NSP16-specific contacts
Shared contacts
NSP10 + NSP16
RNA
NSP16-specific contacts
P0DTC1/7jyy
Shared contacts
NSP10 + NSP16 + RNA
NSP14
P0DTC1/5c8u
NSP14-specific contacts
Shared contacts
NSP10+NSP14
